# Functionally Stable and Phylogenetically Diverse Microbial Enrichments from Microbial Fuel Cells during Wastewater Treatment

**DOI:** 10.1371/journal.pone.0030495

**Published:** 2012-02-07

**Authors:** Shun'ichi Ishii, Shino Suzuki, Trina M. Norden-Krichmar, Kenneth H. Nealson, Yuji Sekiguchi, Yuri A. Gorby, Orianna Bretschger

**Affiliations:** 1 J. Craig Venter Institute, San Diego, California, United States of America; 2 Biomedical Research Institute, National Institute of Advanced Industrial Science and Technology (AIST), Tsukuba, Ibaraki, Japan; 3 University of Southern California, Los Angeles, California, United States of America; 4 Japan Society for the Promotion of Science (JSPS), Chiyoda-ku, Tokyo, Japan; Texas A&M University, United States of America

## Abstract

Microbial fuel cells (MFCs) are devices that exploit microorganisms as biocatalysts to recover energy from organic matter in the form of electricity. One of the goals of MFC research is to develop the technology for cost-effective wastewater treatment. However, before practical MFC applications are implemented it is important to gain fundamental knowledge about long-term system performance, reproducibility, and the formation and maintenance of functionally-stable microbial communities. Here we report findings from a MFC operated for over 300 days using only primary clarifier effluent collected from a municipal wastewater treatment plant as the microbial resource and substrate. The system was operated in a repeat-batch mode, where the reactor solution was replaced once every two weeks with new primary effluent that consisted of different microbial and chemical compositions with every batch exchange. The turbidity of the primary clarifier effluent solution notably decreased, and 97% of biological oxygen demand (BOD) was removed after an 8–13 day residence time for each batch cycle. On average, the limiting current density was 1000 mA/m^2^, the maximum power density was 13 mW/m^2^, and coulombic efficiency was 25%. Interestingly, the electrochemical performance and BOD removal rates were very reproducible throughout MFC operation regardless of the sample variability associated with each wastewater exchange. While MFC performance was very reproducible, the phylogenetic analyses of anode-associated electricity-generating biofilms showed that the microbial populations temporally fluctuated and maintained a high biodiversity throughout the year-long experiment. These results suggest that MFC communities are both self-selecting and self-optimizing, thereby able to develop and maintain functional stability regardless of fluctuations in carbon source(s) and regular introduction of microbial competitors. These results contribute significantly toward the practical application of MFC systems for long-term wastewater treatment as well as demonstrating MFC technology as a useful device to enrich for functionally stable microbial populations.

## Introduction

Presently, wastewater treatment is an energy intensive and expensive process. In the USA over 126 billion liters of domestic wastewater are treated daily at an annual cost of over $25 billion [1]. Therefore decreasing total energy consumption during wastewater treatment is an important goal that can be accomplished through several strategies including: 1) implementing energy efficient equipment and practices; 2) recovering energy during treatment processes; and 3) optimizing treatment methods to minimize overall disposal costs of wastewater effluents and biosolids.

Here we address the use of microbial fuel cells (MFCs) for the degradation of carbon sources in primary clarifier effluents from a conventional wastewater treatment plant. MFC treatment may be utilized to replace or supplement conventional secondary treatment systems and minimize the overall costs associated with aeration, secondary clarification, and secondary sludge treatment. MFC technology exploits biological fermentation and respiratory mechanisms to directly recover energy as electricity during the degradation of organic matter contained in wastewater and/or sludge [2], [3], [4]. Relative to conventional primary and secondary treatment processes, MFC systems also have the benefit of reducing overall operational costs because aeration is not needed [5]. In addition, lower overall sludge volumes can be realized because the growth of secondary biomass is limited under anaerobic MFC conditions [6].

**Figure 1:**
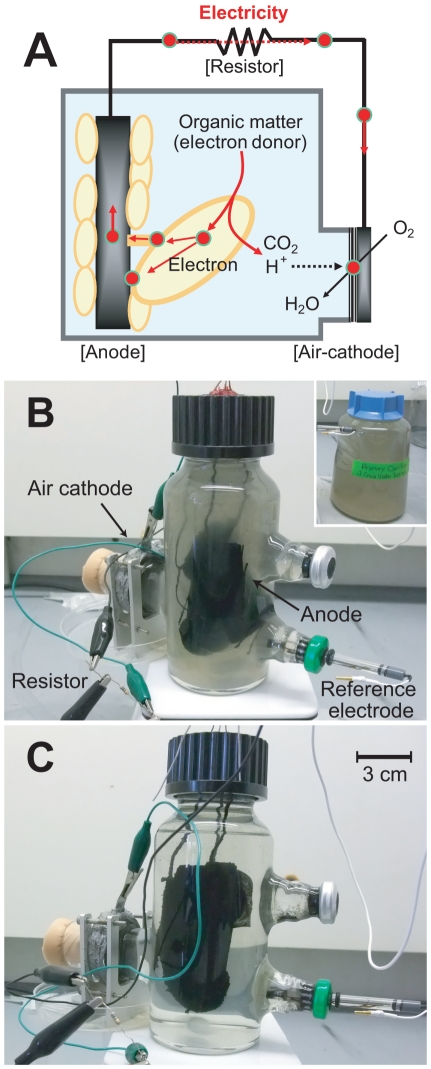
Microbial fuel cell (MFC) used in this study. Schematic diagram of microbial electricity generation in an air-cathode microbial fuel cell (A). The air-cathode microbial fuel cell filled with untreated primary clarifier effluent (shown in inset) (B). The microbial fuel cell after completed treatment (C). Anode and air-cathode were connected with a 750 Ω resistor. A Ag/AgCl reference electrode was used for linear sweep voltammetry.

A MFC reactor physically separates the oxidation and reduction reactions [7]. The biological oxidation of organic matter proceeds in the anode chamber of a MFC under anaerobic conditions. Reducing equivalents (electrons) liberated during the oxidation processes are biologically transferred to a conductive anode electrode where they flow as electrical current across the MFC circuit to the neighboring cathode electrode (Fig. 1A). Protons resulting from the oxidation processes travel by diffusion to the cathode chamber where the terminal reduction reaction consumes the electrons, protons, and a given oxidant. The cathode reduction reaction is typically catalyzed by a noble metal substrate, but biocathodes have also been explored [8]. Both the biological and engineered MFC components influence the total performance (e.g., power density, coulombic efficiency and organic-loading rate) of a MFC system [7], and have been a subject for improvement [6].

Most of the reported evaluations of MFCs have utilized a single carbon source (e.g. acetate, glucose, cellulose), or homogenous effluents, such as synthetic wastewater or industrial wastewater (e.g. brewery waste) to explore the degradation of organic matter and energy recovery as electricity [2]. However, the treatment of municipal primary clarifier effluents is a greater challenge for MFC technology because the clarifier effluents generally contain low concentrations of highly variable organic matters, and at the same time contains highly diverse and temporally variable microbial populations. Consequently, the long-term functional stability of MFC-based wastewater treatment may be severely impacted by the inherent compositional variability of wastewater.

Sustained microbial catalytic activity and metabolic function are the most important factors to consider for long-term wastewater treatment using MFCs. Effective utilization of biological resources for flexible self-organization and self-regulation is needed to treat heterogeneous mixtures of organic and inorganic substrates contained in municipal wastewater. In particular, the microbial community in a MFC must play two critical roles: 1) rapid and complete degradation of various organic compounds through microbial metabolism; and 2) efficient recovery of energy through microbial energy transduction via extracellular electron transport to the anode. Controlling and optimizing these two biological roles is critical to enhance and stabilize MFC performance. To these ends, it is necessary to elucidate microbial function(s) from the enzyme level to the microbial community level.

Here we present the first year-long evaluation of microbial diversity and functionality in a MFC that used municipal primary clarifier effluent as the sole substrate and inoculum source. Microbial functional stability was evaluated by quantifying organic matter degradation and electricity production. Analyses of microbial population dynamics associated with refined statistics were concurrently performed so that we are able to present the first data set that contributes to understanding the population dynamics of a given electricity-generating microbial consortium during long-term MFC operation. Finally, through combined system performance and phylogenetic analyses, we are able to better understand how variable carbon sources and competing microbial communities affect the performance and biodiversity of MFC anode-associated microbial communities.

## Results

### Long-term MFC operation

**Figure 2:**
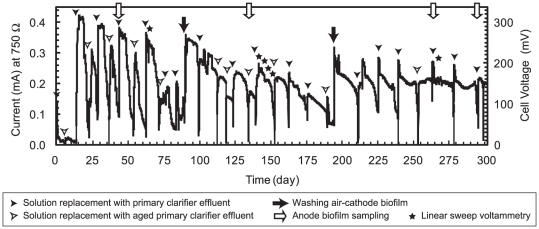
Long-term electricity generation in an air-cathode MFC operating with a 750 Ω external resistance. Filled arrow, removal of cathode biofilm; Open arrow, anode biofilm sampling for DNA extraction; Filled arrowhead, solution replacement with primary clarifier effluents collected on the same day; Open arrowhead, solution replacement with primary clarifier effluents stored at 4°C for 5–10 days; Solid star, linear sweep voltammetry to analyze anode performance.

A single-chamber, air-cathode MFC [9] was used to explore the electrochemical performance and microbial functionality of a wastewater-utilizing MFC system (Fig. 1A–C). The bottle-type MFC was directly inoculated with primary clarifier effluent collected from the North City Water Reclamation Plant (San Diego, USA) (Fig. 1B). Current production was observed after a 13-day lag time and stabilized after two consecutive primary clarifier effluent exchanges (Fig. 2). Subsequently, primary clarifier effluent was collected bimonthly and used for repeat-batch operation with complete replacement of the anolyte after decreasing current generation was observed. After a month of operation under an applied external resistor of 750 Ω, the electric current stabilized at approximately 0.4 mA with 300 mV of the cell voltage. Some differences in current maxima were apparent when the solution was replaced with newly collected wastewater or stored wastewater (at 4°C for up to two weeks) likely due to the lower concentration of BOD contained in the stored samples (Fig. 2).

During the long-term MFC operation, current generation was also impacted by biofilm formation on the cathode surface. After three months of operation, a dense biofilm was visibly apparent at the cathode surface and led to a reduction of current generation due to an overall increase in internal resistance (Fig. 2). To restore previously observed current generation and internal resistance measurements, the cathode was mechanically treated to remove the attached biofilm. This treatment occurred twice during the 300 day operational period, the first time on day 88 and again at day 192. Subsequently, stable current production was observed to be approximately 0.25 mA, and the repeat-batch cycles were 8–15 days depending on the condition of primary clarifier effluents.

### Organic matter degradation

The purpose of repeat-batch cycling was to observe the reproducible organic matter degradation as measured by a decrease in turbidity, removal of chemical oxygen demand (COD), biological oxygen demand (BOD), and inorganic compounds. Although turbidity of the original sample was high, the anolyte solution was almost completely clear after the 8–13 day residence time (Fig. 1C). Organic matter as determined by COD also consistently decreased with current generation (Fig. S1). Our results show an average of 86±2% of COD was removed in a typical cycle. Organic matter as determined by BOD also significantly decreased with 97% of BOD being removed in a single batch (Table 1). These results suggest that the depletion of biodegradable organic chemicals coincided with the decrease in current generation. Electron recovery was calculated as coulombic efficiency, which was found to be stable at 26±1% over the repeat-batch cycles. That is, approximately 75% of electrons associated with the COD degradation of each sample were consumed by competing reactions such as oxygen respiration, anaerobic respiration with soluble electron acceptors such as sulfate or nitrate reduction, and/or biosynthesis. During long-term operation we did not visibly observe any liquid displacement or significant volume changes with gas production in the reactor suggesting that fermentation, methanogenesis and/or hydrogenesis could not occur at high rates.

**Table 1:** Chemical composition of primary clarifier effluent and MFC effluent.

	Primary clarifier effluent^b^	MFC effluent^c^	Removal ratio (%)
COD (mg/L)^a^	263.3	36.8	86
BOD (mg/L)^a^	181.4	6.2	97
TSS (mg/L)^a^	116.0	22.3	81
Turbidity (NTU)	88.7	41.2	54
Nitrate-N (mg/L)	0.03	0.03	0
Nitrite-N (mg/L)	0.15	<0.01	>93
Ammonia-N (mg/L)	32.4	26.2	19
Sulfate (mg/L)	262.1	218.1	17
Heavy metal conc. (µg/L)			
Fe	1688	1027	39
Sr	1120	1010	10
Al	717	120	83
Mn	125	129	−3
Cu	82	23	72
Zn	79	33	58
Mo	12	3	75
Cr	6	1	80

a COD = chemical oxygen demand, BOD = biological oxygen demand, and TSS = total suspended solid.

b Primary clarifier effluent was collected on day 225 and was directly added to the MFC.

c The MFC effluent was collected on day 239.

Other components of the primary clarifier effluent sample and treated MFC anolyte are compared in Table 1. These comparisons show that potential anaerobic electron acceptors such as sulfate and nitrite were slightly reduced during the MFC batch cycle. These data suggest that alternative electron acceptors existed in the primary clarifier wastewater but were not used as the preferential terminal electron acceptors during anaerobic respiration. Oxygen is known to permeate the air-breathing cathodes used in these experiments. However, thick biofilms were observed at the cathode surface (data not shown) and were likely responsible for the removal of oxygen at the cathode surface and maintenance of anaerobic conditions at the anode. The concentrations of several inorganic heavy metals (except for manganese and strontium) were significantly decreased in the process; however, the oxidation states of these metals were not quantified so it is unknown if the metals served as electron acceptors or if abiotic adsorption contributed to these observed values. The conductivity (1.7 mS/cm) and pH (7.57) of the MFC anolyte were unchanged from their values seen in the raw primary clarifier effluent, and were stable throughout each cycle.

High pressure liquid chromatography (HPLC) analysis of the raw primary clarifier effluent and treated MFC anolytes revealed that various organic chemicals were nearly completely degraded in the MFC process (Fig. S2). While there was a clear decrease in number and amounts of organics, many of the peaks could not be matched to known standards of volatile fatty acids due to the complex nature of the raw sample. The publically available, North City Water Reclamation Plant (NCWRP) annual report indicates that approximately 30 mg/l of n-hexane extractable material (organic solvents) and 8 mg/l of methylene blue active substances (MBAS) (surfactants) were present in the primary clarifier effluent [10]. It is possible that many of the unidentified peaks in the HPLC chromatographs may be related to organic solvents and/or surfactants.

### Electricity production

**Figure 3:**
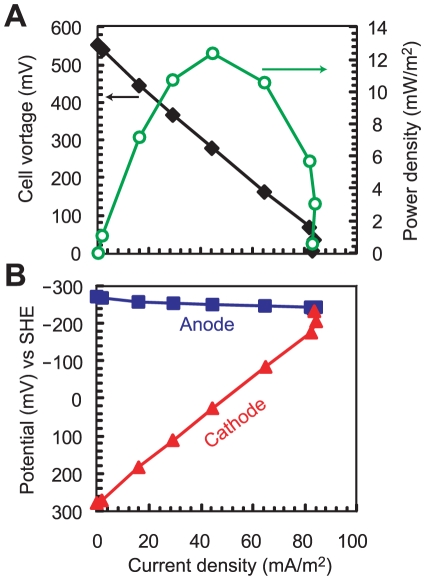
Power curve and polarization curves in the primary clarifier effluent-fed MFC. Power curve (A) and anodic and cathodic polarization (B) measured from the MFC after stable current production was observed on day 32. Anode potential (solid square), cathode potential (solid triangle), and cell voltages (solid diamond) were measured at various external resistances and plotted versus current density normalized to the projected anode surface area. Reactor power performance is represented by power density per anode surface area (open circle).

Performance of MFC systems are commonly evaluated in terms of power and current densities [11]. Electricity production was monitored as current versus time, but additional electrochemical measurements were performed to thoroughly characterize the MFC system. On day 32, anodic, cathodic and whole electrochemical cell polarization curves were determined by using a graded series of external resistors (Fig. 3). These curves showed an open circuit cell voltage of 550 mV, open circuit anode potential of −270 mV vs Standard Hydrogen Electrode (SHE), and open circuit cathode potential of +280 mV vs SHE. The maximum power density per projected anode surface area was 12.4 mW/m^2^ (with 750 Ω of external resistance), while the maximum power output per reactor volume was 0.3 W/m^3^. The limiting current density was 84 mA/m^2^ with 10 Ω of external resistance (Fig. 3A). The anodic and cathodic polarization curves apparently revealed that reactor performance was cathode limited (Fig. 3B), suggesting that the cell polarization curve did not reflect the available microbial biocatalytic activity at the anode [9], [12].

**Figure 4:**
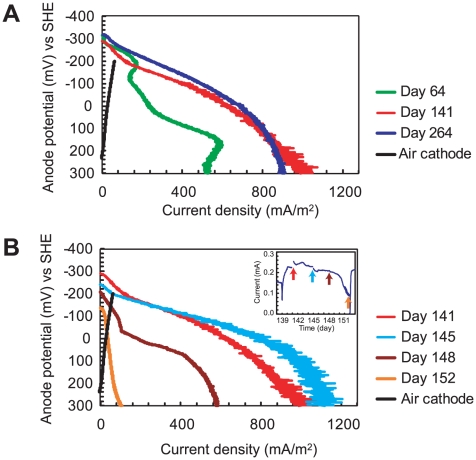
Anode polarization curves in the primary clarifier effluent-fed MFC determined by linear sweep voltammetry. The anode polarization curves during the enrichment process of electricity generating mixed community (A). The anode polarization curves during a single batch operation from day 139 to day 151, current generations are shown in the inset (B). Cathode polarization curve (black line) was determined on day 108.

In order to better analyze the microbial current generating properties, the anode polarization curves were determined by linear sweep voltammetry (LSV) (Fig. 4). Using a potentiostat, the anode potential was varied from the open circuit anode potential to +300 mV vs SHE at a scan rate of 0.5 mV/sec, allowing measurement of anodic activity independent of cathode limitations [12]. The anode polarization curves on day 64 (Fig. 4A) revealed a limiting current density of approximately 600 mA/m^2^. After another 3 months of operation, the limiting current density improved to 1,000 mA/m^2^ (day 141), indicating that electricity generating microorganisms were further optimized during the repeat-batch operation. However, the limiting current density did not increase again after another 100 days of operation (measured on day 264), suggesting that electricity generating performance had stabilized between day 141 and day 264. This current density of 1,000 mA/m^2^ represents the limiting anodic biocatalytic activity and may be a function of low ionic conductivity in the anolyte (1.7 mS/cm), increasing the ohmic losses in the system; and/or low substrate concentration at the biofilm surface (the COD was 260 mg/L) as a result of diffusion limitations.

### Correlation between anode biomass and electricity production

Each repeat-batch cycle began with new and different suspensions of chemical compounds and microbes. The anode polarization curves were also measured during the representative single batch-feed from day 139 to day 152 (Fig. 4B). The polarization curves revealed similar trends through the initial 6 days, while the curves after day 9 showed an abrupt decrease in maximum current density and a more electropositive open circuit anode potential. These changes affirm the drop in current production as a result of the reduction of available organic matter.

The relationship between each new microbial cell suspension and the observed patterns of electricity production and organic matter degradation was also examined. The suspended cell concentrations decreased during the residence time associated with each batch cycle. During a representative cycle, it was found that the suspended cell counts decreased from 7.5×10^7^ cells/ml at day 139 to 1.2×10^7^ cells/ml at day 152, suggesting that planktonic microbial cells were not contributing to organic matter degradation or electricity generation in the MFC. This is also visibly evident in the turbidity changes shown in Fig. 1B and 1C. Scanning electron microscope (SEM) observations of the anode surface at day 152 clearly revealed a dense biofilm constructed on the surface (Fig. S3). The biomass density of the biofilm was determined as total protein concentration per anode surface area, resulting in 1.25 mg-protein/cm^2^ on day 152. In order to analyze normalized biocatalytic activity of the anode biofilm, the per-biomass electron-donating rate was calculated to be 52.4 µmol-electron g-protein^−1^ min^−1^.

These data, when taken together, suggest that microbial growth primarily occurred at the electrode surfaces and that the anode consortium was primarily responsible for organic matter degradation and current production in the MFC.

### Phylogenetic composition

To analyze the microbial community composition dynamics of the anode biofilm, we constructed 16 S rRNA gene clone libraries of anode biofilm samples collected at day 44 (W1), day 133 (W2), day 263 (W3), and day 294 (W4). Clone libraries prepared from raw primary clarifier effluent samples collected at day 14 (PC1), day 152 (PC2), and day 304 (PC4) were also analyzed because these samples served as carbon and inoculum sources for each repeat-batch cycle.

**Figure 5:**
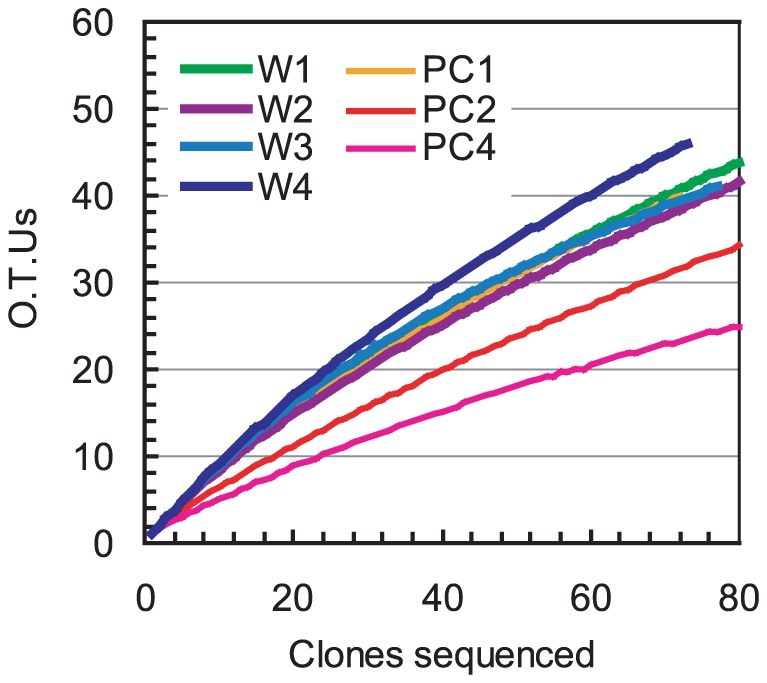
Rarefaction curves for the different phylotypes obtained from 16 S rRNA gene clone libraries. The raw primary clarifier effluent samples were collected at day 14 (PC1), day 152 (PC2), and day 304 (PC4). The anode biofilm samples were collected on day 44 (W1), day 133 (W2), day 263 (W3), and day 294 (W4).

The rarefaction-curve analysis for the MFC anode and primary clarifier samples showed that the anode biofilm community at any given time was more diverse than the communities of primary clarifier effluents (Fig. 5). This result indicates that counts of phylotypes in the anode biofilm were not reduced throughout the year long enrichment process. The Shannon's diversity index, Simpson diversity index, and Chao-1 richness also suggest this trend (Table 2).

**Table 2:** Diversity statistics and recovery of bacterial phyla from MFC anode biofilms and primary clarifier effluents.

Source	Anode biofilm				Primary clarifier effluent		
Library ID	W1	W2	W3	W4	PC1	PC2	PC4
Sampling day	44	133	263	295	14	152	304
Shannon's Index	3.5	3.4	3.5	3.6	3.3	2.8	2.0
Simpson Diversity Index (1 - D)	0.96	0.94	0.96	0.97	0.94	0.80	0.66
Number of clones sequenced	88	84	77	73	72	146	91
Number of O.T.U. (99% cutoff)	47	43	41	46	40	54	27
Chao1 Richness	228±65	203±59	77±15	119±27	96±22	180±42	45±10
Sørensen's similarity coefficients							
W1		**0.18**	0.11	0.11	0.09	0.06	0.11
W2			**0.21**	0.14	0.12	0.10	0.14
W3				**0.32**	0.03	0.00	0.00
W4					0.00	0.02	0.03
PC1						**0.28**	**0.21**
PC2							**0.20**

**Figure 6:**
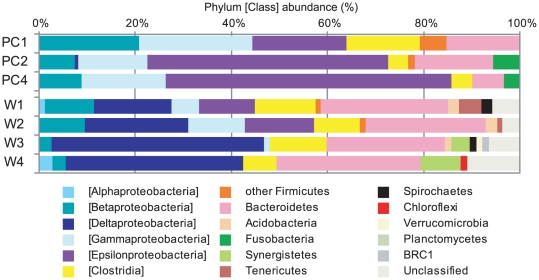
Phylum level taxonomic distribution of 16 S rRNA community profile. The taxonomic profiles were analyzed for the original primary clarifier effluents (PC1, PC2, PC4) and the MFC anode biofilms (W1–W4). Phylum *Proteobacteria* and *Firmicutes* are divided into class level taxonomies.

Results from the seven different 16 S rRNA clone library analyses are shown in Fig. 6 and summarize the phylogenetic affiliations to taxa at the phylum or class level. The analyses indicate that all clones were affiliated with the domain *Bacteria*. The relatively high abundant phyla in the 16 S rRNA clone libraries of both the anode biofilm and primary clarifier effluents were *Proteobacteria*, *Firmicutes*, and *Bacteroidetes*. Those phylotypes are considered as relatively abundant species in the microbial communities. Of the *Proteobacteria*, members of the class *Deltaproteobacteria* were abundant in the anode biofilm samples and significantly increased in abundance throughout the long-term MFC operation. In contrast, those of the *Betaproteobacteria, Gammaproteobacteria*, and *Epsilonproteobacteria* decreased in abundance within the anode biofilm samples even though these members were abundant in every characterized primary clarifier effluent sample. These results indicate that deltaproteobacterial species are important for electricity generation in the anode biofilm. Furthermore, the frequency of the phylum *Bacteroidetes* in the anode biofilm was slightly higher than that of primary clarifier effluents, suggesting that *Bacteroidetes* could also be important for efficient biofilm function.

**Figure 7:**
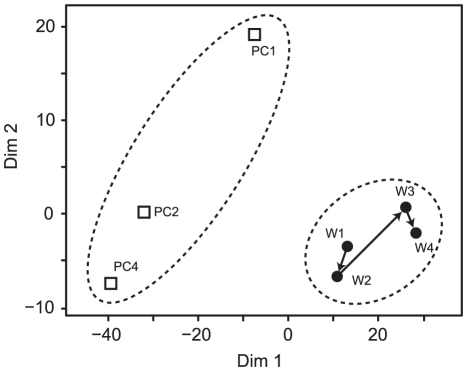
Multidimensional scale plot comparing bacterial communities based on genus level taxonomy. Open square, the bacterial communities of original primary clarifier effluents (PC1, PC2, PC4); Filled circle, the bacterial communities of anode biofilm enriched in the primary clarifier effluent-fed MFC (W1–W4).

In order to further compare the microbial communities, a multidimensional scale plot (MDS) was created based on genus level taxonomy (Fig. 7). The plot suggests that the microbial communities associated with primary clarifier effluents were more diverse than those of the anode biofilm, and there was a significant difference between those two groups. Sørensen's similarity coefficient was also used to statistically compare the similarity of these two types of microbial communities. The summary of Sørensen's similarity coefficients among the seven microbial communities based on operational taxonomy unit (OTU) at 99% cut off is shown in Table 2. These results indicate that the functionally enriched anode community varies slightly with time, but is significantly different than the planktonic populations that challenge the existing biofilm with each repeat-batch cycle.

In the early phases of MFC operation (W1 and W2), the anodic microbial communities had slight similarities to the microbial communities of the primary clarifier effluents. These results suggest that some of the initial bacteria introduced to the MFC reactor attached and then established the electricity-generating microbial community in the anode biofilm. Furthermore, the data indicate that the established biofilm was not susceptible to invasion by new microbial populations introduced with the primary clarifier repeat-batch anolyte replacements.

### Electricity-generating bacteria

Table 3 shows the abundant phylotypes, containing more than 5 clones, from the anode electricity-generating biofilm samples W1–W4. Large portions (∼50%) of the biofilm clones were categorized into the minor phylotypes, which are summarized in Table S1.

**Table 3:** Abundant phylotypes obtained from the enriched electricity-generating consortia^a^.

Phylum-Class Phylotype	No. of clone in the library				% Database match (Accession No.)
	W1	W2	W3	W4	
***Proteobacteria - Deltaproteobacteria***					
W1_B02	11	-	-	-	98% *Geobacter* sp. Ply1 (EF527233)
W2_M05	-	13	-	-	99% *Geobacter lovleyi* SZ (CP001089)
W3_M01	-	1	6	3	96% *Geobacter lovleyi* SZ (CP001089)
W2_A03	-	1	7	8	99% *Desulfuromonas acetexigens* (U23140)
W3_A19	-	-	4	2	99% *Desulfobacter postgatei* DSM 2034 (AF418180)
W3_O19	-	-	3	3	99% *Desulfocapsa thiozymogenes* (X95181)
W3_O03	-	-	2	3	91% *Desulfobulbus rhabdoformis* Mic5c02 (AB546248)
***Proteobacteria - Betaproteobacteria***					
W1_F12	3	2	-	-	100% *Acidovorax* sp. PPs-5 (FJ605421)
***Proteobacteria - Gammaproteobacteria***					
W2_C01	-	5	1	-	100% *Pseudomonas* sp. SMT-9 partial (AM689953)
***Proteobacteria - Epsilonproteobacteria***					
W1_J08	9	10	-	-	100% *Arcobacter cryaerophilu*s (U34387)
***Bacteroidetes***					
W2_I23	2	1	3	3	88% *Ruminofilibacter xylanolyticum* S1 (DQ141183)
W1_B10	4	3	1	1	87% *Cellulophaga tyrosinoxydans* EM41 (EU443205)
W1_J04	4	-	1	-	93% *Parabacteroides goldsteinii* JCM13446 (EU136697)
W2_C17	-	3	2	-	90% *Prolixibacter bellariivorans* F2 (AY918928)
W4_O10	-	-	4	3	90% *Cytophaga fermentans* (M58766)
W3_A07	-	-	4	2	88% *Cytophaga fermentans* (M58766)
***Firmicutes - Clostridia***					
W1_H18	3	3	1	-	97% *Fusibacter* sp. SA1 (AF491333)
***Synergistetes***					
W3_A23	-	-	3	4	90% *Aminomonas paucivorans* (AF072581)
**Others** b	52	42	35	41	
Total clone	88	84	77	73	

a Abundant phylotype was defined as a phylotype containing more than 5 clones within four clone libraries.

b For a list of other phylotypes (minor phylotypes), refer to the supplementary materials (Table S1).

#### *Deltaproteobacteria*

As mentioned above, *Deltaproteobacteria* are potentially important bacteria for extracellular electron transfer in the anode biofilm, however, the dominant phylotypes within this class changed over time. During the early stage of MFC operation (day 44, W1 clone library) the *Deltaproteobacteria* were primarily comprised of a phylotype W1_B02, which is closely related to *Geobacter* sp. Ply1. However, during the following stage (day 133, W2 clone library) the dominant phylotype was W2_M05, which is more related to *Geobacter lovleyi* SZ. Over 200 days of operation, the matured biofilm samples represented in W3 (day 263) and W4 (day 294) clone libraries showed that the dominate phylotypes at the early stages had diminished, and another five abundant phylotypes were apparent in the class *Deltaproteobacteria*. The phylotype W2_A03, which appeared first in the W2 clone library, subsequently became dominant as the biofilm continued to mature, was found to be closely related to *Desulfuromonas acetexigens*.

#### *Epsilonproteobacteria*

A phylotype W1_J08, which is closely related to *Arcobacter cryaerophilus*, was highly abundant in only the early stages of anode microbial community development (Table 3). This phylotype was also abundantly present in the primary clarifier effluent (Table S2), suggesting that the bacterium was simply introduced from the wastewater but did not thrive at the anode surface during prolonged electricity generating conditions.

#### *Bacteroidetes*

The phylum *Bacteroidetes* was also abundant in the anode biofilm and various abundant phylotypes were observed throughout the long-term MFC operation (Table 3). Interestingly, the *Bacteroidetes* phylotypes that were found in the anode-associated consortia were not observed in the raw primary clarifier effluent (Table S2); furthermore, the phylotypes were not closely related to isolated *Bacteroidetes* strains (Table 3).

## Discussion

The present study successfully investigated the performance and phylogenetic diversity of an MFC community fed solely with raw primary clarifier effluent from a municipal wastewater treatment plant for over 300 days and 31 solution changes (Fig. 2). Despite the regular changes in organic matter composition [10] and repeated exposure to new microbial populations (Table S2), the reported MFC system was able to continuously generate a current of approximately 0.25 mA and degraded the chemical constituents associated with primary clarifier effluents with reproducibility (Fig. 2). These results suggest that the MFC can be a robust tool for wastewater treatment and for selecting functionally stable microbial communities from diverse and dynamic inoculum sources. The anodic biocatalytic activity that is one of the most important parameter for developing electrochemical fuel cells was also reproducible after day 141, yielding a current density of approximately 1,000 mA/m^2^ (Fig. 4A), which if modified may lead to useful energy recovery at a treatment plant scale.

To consider the implementation of MFC technology in a wastewater treatment facility, it is important to demonstrate that removal rates and electricity generation are not affected by seasonal changes in wastewater effluents or the variability of chemical compounds and concentrations. This can only be proven by long-term MFC evaluations of the microbial community dynamics and resulting current production and COD removal rates. Here we present a comprehensive data set that contributes to a better understanding about how electrogenic microbes respond to real wastewater conditions. Using this knowledge, we can begin to make system design improvements to increase current densities, bioaugment or pre-seed anode associated communities for rapid MFC enrichment, and ultimately speed COD degradation rates. Although an 8–13 day treatment time is not acceptable for practical application to wastewater treatment, we believe the COD removal rates will significantly increase with design modifications to the system, such as lowering internal resistance and improved cathode catalysts, and using our enriched electricity-generating microbial communities as anode catalysts.

Several reports have recently appeared in the literature exploring MFC performance during treatment of primary clarifier effluents [13], [14], [15], [16]. Di Lorenzo *et al.* described the phylogenetic composition of an electricity-generating microbial community associated with a MFC anode by using denaturing gradient gel electrophoresis (DGGE) based on 16 S rRNA gene fragments. However, the microbial community was only analyzed as a function of different anode surfaces within the system and not as a function of time, wastewater inoculum sources, or MFC performance [13]. Therefore, there is much to understand about the microbial populations responsible for catalyzing rapid degradation of organic compounds and electricity production, and how microbial populations assemble and functionally maintain in MFCs. Here, we conducted electrochemical analyses and clone analyses based on 16 S rRNA gene sequences to achieve a more detailed understanding about the composition of the microbial population responsible for electricity production and the degradation of organic compounds, and how the population is correlated to biological and chemical heterogeneity of the influents throughout long-term operation. In addition, we periodically compared the anode biofilm to the introduced microbial community from the primary clarifier effluents to discern how the community diverged from the planktonic inoculum and substrate sources.

Three distinct microbial communities existed in the MFC system: anode biofilm (anaerobic metabolism), cathode biofilm (aerobic/facultative metabolism), and suspended (planktonic) cells (facultative metabolism) [17], [18]. Each population may contribute to overall system operation in terms of organic degradation rates and electricity generation. During each batch cycle, the suspended cell density decreased by one-sixth, suggesting that planktonic cells were not significantly contributing to direct electron transfer or COD/BOD removal. Further, our repeat-batch approach decreased the accumulation of mediators (electron shuttles) in the anolyte so the presence of such compounds would need to be within the biofilm matrix, or in very close proximity to the electrode surface in order to contribute to the observed current densities.

Although it is known that the air-cathode we used allows the diffusion of oxygen into the MFC system [19], [20], a robust biofilm was found to thrive at this surface. It is likely that this biofilm was responsible for capturing oxygen, which was important to maintaining the anaerobic conditions required for efficient electricity generation at the anode. The success of this process can be noted through the presence of strict anaerobes that thrived at the anode surface, but were not present in high abundance in the primary clarifier effluents. Consequently, it can be inferred that the anode biofilm was mainly responsible for electricity production through direct extracellular electron transfer from biofilm constituents to the anode surface (Fig. 1A).

In a previously reported study using a cellulose-fed MFC, the diversity of the anode microbial community decreased with time while system performance remained stable [21]. However, the electricity-generating microbial community in the primary clarifier effluent-fed MFC did not show a decrease in diversity even though the system operation remained stable (Fig. 5 and Table 2). This result indicates that a complex microbial community was necessary to degrade diverse and variable organic substrates. Interestingly, the introduction of new microbes with each repeat-batch cycle did not appear to impact the electricity-generating biofilm. Throughout MFC operation the anode-associated population was found to change, but each generation was more closely related to its surface-attached predecessors and was very different from the microbial populations that were introduced with the new primary clarifier effluents.

Although the anode-associated microbial community was still diverse after the enrichment process, the taxonomic distribution of the electricity-generating microbial community clearly reached a stable population (Fig. 7). The early stage microbial community was slightly affected by the introduction of primary clarifier microbes including *Acidovorax* sp. and *Arcobacter* spp., which were present in relatively high abundance in the primary clarifier effluent (Table S1). Over time, the community shifted to a more “conventional” anode-respiring population (Table 3) including *Geobacter* and *Desulfuromonas* spp., indicating that a longer-term enrichment process facilitated the adaptation to the electrode-reducing conditions while oxidizing complex heterogeneous substrates in the primary clarifier effluent.

The enriched electricity-generating anode microbial community was mostly comprised of the class *Deltaproteobacteria* and the phylum *Bacteroidetes* (Fig. 6), which have been frequently observed as dominant taxa in both sediment MFCs [22], [23] and in MFC reactors fed with industrial wastewaters [24], artificial wastewater [17], or with defined chemicals such as acetate and glucose [9], [18], [25]. The class *Deltaproteobacteria* includes various dissimilatory solid metal reducing bacteria [26], some of which are also reported as electricity generators in MFC systems [27], [28], [29].

The *Geobacter* spp. are well-known electricity generating bacteria [27], [28], indicating that the phylotypes observed within the anode-associated community are likely playing an important role in electricity production. Recently, many *Geobacter* strains have been observed as part of electricity-generating microbial communities, especially in acetate-fed MFC anodes [18], [25], [30], suggesting that the *Geobacter* strains are oxidizing acetate during anode respiration. Various *Geobacter* strains including *Geobacter metallireducens* can also utilize a wide variety of electron donors including toluene and benzoate [31], suggesting that the abundant *Geobacter* strains in the described system may also be playing a role in the oxidation of more complex primary effluent substrates.

In the highly enriched electricity-generating anode microbial communities (W3 and W4), four other *Deltaproteobacteria* phylotypes closely related to genera *Desulfuromonas*, *Desulfobacter*, *Desulfocapsa*, and *Desulfobulbus* were also observed (Table 3). The most abundant phylotype W2_A03 in the mature biofilm was found to be closely related with *Desulfuromonas acetexigens*, which has been reported as a solid iron/electrode reducer [32]. The strain has also been observed in electrically active anode biofilms in sediment MFCs [22], [23], and may therefore be contributing to electricity generation in our system. Other prevalent phylotypes were closely associated to *Desulfocapsa* and *Desulfobulbus*, *which* have both been previously reported as potential electrode reducers [22], [29], [33].

The prevalence of different electricity generating phylotypes in our primary clarifier MFC implies that several species within the *Deltaproteobacteria* class were syntrophically cooperating to produce electricity from the wide varieties of chemical compounds in the primary clarifier effluents. Interestingly, the dominant *Deltaproteobacteria* species changed with time, but the electricity generating performance and chemical oxidation rates remained stable. This phenomenon clearly suggests that the anode-associated microbial population can be functionally maintained for the treatment of primary clarifier effluents and sustain energy recovery in the process.

We hypothesize that the phylotypes associated with *Deltaproteobacteria* species were primarily responsible for direct electricity production; however the other highly abundant anode-associated phylotypes were closely related to the phylum *Bacteroidetes* and observed in all stages of anode biofilm enrichment. While the phylum *Bacteroidetes* has mainly been described as a fermentor in the human gut [34], Shimoyama *et al.* recently demonstrated that *Bacteroidetes* was an abundant phylum correlated with electricity generation from artificial wastewater treatment in a continuous-flow cassette-electrode MFC [17]. However, our phylotypes were not closely related to the other previously reported *Bacteroidetes* strains including the clone CE38 abundantly observed in the cassette-electrode MFC. These results suggest that MFC enriched *Bacteroidetes* species may possess diverse functional traits and may thus represent an interesting phylum worthy of further study. To address the potential roles of these dominant phylotypes classified to *Bacteroidetes* within our MFC system, we will attempt to isolate these strains and analyze their genomic and functional characteristics in future work.

Other less dominant phylotypes observed in the anode-associated biofilm were very diverse and functionally unknown (Table S1). Some phylotypes could be contaminants from the primary clarifier effluent (Table S2), while others could be contributing to the degradation of various types of organic chemicals. The long-term survival of these less abundant phylotypes suggest that they have a functional role in organic compound degradation and perhaps even within extracellular electron transfer.

In summary, while MFC performance was very reproducible, the phylogenetic analyses of anode-associated electricity-generating biofilms demonstrated that the microbial populations fluctuated temporally while maintaining high biodiversity throughout the year-long experiment. These results suggest that MFC operation induces a self-optimizing process toward functional performance from diverse, heterogeneous microbial communities, and therefore can be used to reproducibly select for functional microbial communities regardless of carbon source. These results contribute significant knowledge toward the practical application of MFC systems for long-term wastewater treatment, while demonstrating the utility of MFCs for enrichment of functionally stable microbial populations capable of organic compound degradation and extracellular electron transfer.

Additional impact of the reported results is provided by the ability to observe the metabolic activities and energy transduction within a complex consortium. This has significant benefits to the field of microbial ecology and may yield insight into numerous geochemical cycles that involve biological transformations of extracellular material, such as iron and manganese oxides. Future studies will apply metagenomics and metatranscriptomics to the anode biofilm to describe gene expression profiles associated with carbon metabolism and extracellular electron transfer during the degradation of complex organic substrates. Gene expression data will further elucidate metabolic networks and energy transduction in complex consortia.

## Materials and Methods

### MFC configuration and operation

A single-chamber, air-cathode MFC was used for municipal sewage wastewater treatment with power generation. The MFC was a bottle-type reactor (350 ml in capacity), with two joined anode electrodes made of carbon cloth (7 cm×3 cm, or 84 cm^2^ total projected surface area per reactor; TMIL, Japan) [9]. The air-cathode was made with a 30 wt% wet-proofed carbon cloth (type B-1B, E-TEK) coated with platinum (0.5 mg/cm^2^), Nafion, and PTFE as described elsewhere [20]. The air-cathode was placed at the side port, providing a total projected cathode surface area (one side) of 4.9 cm^2^.

After sterilization of the fully assembled MFC, the chamber was filled with municipal wastewater collected from the primary clarifier at the North City Water Reclamation Plant (San Diego, USA) without any pretreatment except the mechanical removal of grit, rags and scum. The sole inoculum source consisted of those microorganisms present in the primary clarifier effluents. The MFC was gently mixed with a magnetic stirrer, and incubated at room temperature (22°C±3°C) throughout the duration of testing.

The anode and cathode electrodes were connected with an external resistor of 750 Ω. Cell voltages across the resistor were recorded every 30 min using a voltage recorder (GL200A, Graphtec) and the corresponding electric current was calculated using Ohm's law (V = IR). When the electric current decreased due to depletion of the organic matter in the wastewater, the anode solution was fully discarded and the reactor was refilled with either the fresh wastewater collected that day or with aged wastewater stored at 4°C. This repeat-batch process occurred twice monthly with fresh wastewater, and weekly with aged wastewater, for 300 days. Each wastewater sample introduced to the reactor included the naturally occurring microorganisms and various chemical compounds, no filtration or additional pretreatment was conducted. On days 88 and 195, the biofilm formed on the cathode surface was mechanically removed to recover the cathode performance.

### Polarization analyses

To obtain polarization and power density curves, an Ag/AgCl reference electrode (+200 mV vs SHE, RE-5B, BASi) was placed in the side port of the MFC. The external resistance across the circuit was then changed stepwise from 3.3 kΩ to 10 Ω and the cell voltage, the anode potential, and the cathode potential were recorded after they had stabilized over a period of at least 7 min [9]. Current density per projected anode surface area was calculated from the voltage measured across the known resistor. Power density was calculated as the product of current density and the cell voltage.

In order to obtain anode polarization curves without cathodic reaction limitation, linear sweep voltammetry analyses were conducted using a potentiostat (Reference 600™, Gamry) [12]. The anode potential was swept from open circuit anode potential to +300 mV vs SHE at a scan rate of 0.5 mV/sec and the corresponding anodic current, resulting from the active biofilm, was recorded.

### Chemical analyses

Chemical oxygen demand (COD) was determined using a potassium chromide assay according to the manufacturer's instructions (Orion CODHP0, Thermo Scientific). Coulombic efficiency, CE (%), was calculated as CE = C_p_/C_th_×100, where C_p_ (C) is the total charge passed during a single batch, and C_th_ (C) is the theoretical amount of charge allowable from a complete COD decrease (assuming that reducing one mole of oxygen requires the transfer of four electrons). Biological oxygen demand (BOD), total suspended solid (TSS), turbidity, nitrate-N, nitrite-N, ammonium-N, sulfate, and heavy metal concentrations were determined in accordance with US EPA and state of California requirements by CRG Marine Laboratories, Inc (Torrance CA, USA). Conductivity of the solution was determined by portable pH/ORP/DO/ionic meter (Orion 1215000, Thermo Scientific). Acetate and other volatile fatty acids were determined using a high-pressure liquid chromatography (HPLC) machine equipped with DI detector (Agilent 1200 series) and a packed C18 column (Epic Polar, ES Industries). The eluant was 50 mM phosphoric acid (pH 1.87) at a flow rate of 1.0 ml/min.

In order to determine the total bacterial cell density on the anode surface, part of the anode (7 mm×7 mm) containing cells was removed from the MFC (n = 3). Total protein was extracted from the electrodes as described elsewhere [9], [28]. Bacterial cell concentrations in the solution were also determined by direct cell counts. Cells were stained with 2 mg/L of 4,6-diamidino-2-phenylindole (DAPI) for 5 min then observed using an AX10 fluorescence microscope (Carl Zeiss).

### Scanning electron microscopy (SEM)

A small portion of carbon cloth was collected from the anode, fixed with 1.25% gultaraldehyde, dehydrated using a graded series of ethanol solutions, and dried using a critical point drier (Autosamdri 815, Tousimis) [35]. The specimen was coated with Pt/Pd and imaged at 2 kV on a LEO 1540XB Field Emission SEM (Carl Zeiss SMT AG). The imaging was conducted at the Western Nanofabrication Facility, University of Western Ontario (Canada).

### PCR amplification, cloning and sequencing of 16 S rRNA gene fragments

Total DNA was extracted from the biofilm associated with the carbon cloth anode or from the suspended cells in the primary clarifier effluent. All DNA extractions were performed using the UltraClean® Soil DNA Isolation Kit (MO bio) according to manufacturer instructions, which employed physical cell disruption. PCR amplification of 16 S rRNA gene fragments was performed using Taq DNA polymerase (ExTaq, Takara) with universal primers U27f (5′-AGAGTTTGATCCTGGCTCAG-3′) and U1492r (5′-GGTTACCTTGTTACGACTT-3′) [36]. The amplification conditions were as follows: an initial step of 94°C for 3 min, 25 cycles consisting of 94°C for 30 sec, 55°C for 30 sec and 72°C for 90 sec, and a final elongation step at 72°C for 10 min. Amplified fragments were ligated into a pGEM-T vector (Promega) and cloned into *Escherichia coli* JM109 competent cells. PCR-amplified 16 S rRNA gene fragments were recovered by PCR using primers M13f and M13r (the primers targeted the pGEM-T vector sequences flanking the insertion), then sequenced by ABI 3730xl sequencers using primer U907r (5′-CCGYCAATTCMTTTRAGTTT-3′) [37]. The nucleotide sequences reported in this paper have been deposited in the GSDB, DDBJ, EMBL and NCBI nucleotide sequence databases under accession numbers HQ688300 to HQ688420 for the primary clarifier effluents, and HQ688421 to HQ688596 for the anode biofilm.

### Phylogenetic analyses

Sequences of partial 16 S rRNA genes determined in this study were aligned to each other using CLC genomics work bench version 3.6.5 (CLC bio), and assigned to phylotypes (classified as an operational taxonomic unit, >99% cut-off). Database searches for related 16 S rRNA gene sequences were conducted using the BLAST program [38]. Checks for chimeric sequences and a multidimensional scale (MDS) plot were conducted using JCVI 16 S/18 S small sub-unit analysis pipeline. A rarefaction analysis was conducted using the Analytic Rarefaction program [21]. Chao1 richness was calculated using web-based software (http://www2.biology.ualberta.ca./jbrzusto/rarefact.php). A Shannon's index, Simpson diversity index, and Sorensen similarities among the bacterial communities were calculated using Estimate S [39].

## Supporting Information

Figure S1 **Typical batch cycle of current generation and COD concentrations in the primary clarifier effluent-fed MFC.** Thin black line, electric current (mA); Thick red line, accumulated electron production expressed as ‘mM equivalent (eq.)’ calculated as the number of total electrons passing across the circuit from the available COD in solution; solid square blue line, total COD (mg/L) in solution.
(EPS)

Figure S2 **HPLC chromatographs of wastewater samples before and after MFC treatment.** Organic compounds were detected at wavelengths of 210 nm (A, B) or 254 nm (C, D). The untreated primary clarifier effluent chromatographs indicate the presence of several different compounds (A, C), most of which were no longer present in the MFC treated samples (B, D).
(EPS)

Figure S3 **FE-SEM images for anode biofilms adhering onto carbon cloth anodes (day 152).** Bar in panel A is 100 µm, bar in panel B is 10 µm, and bars in panel C and D are 2 µm.
(EPS)

Table S1 **All phylotypes obtained from the enriched electricity-generating consortia.**
(XLS)

Table S2 **All phylotypes obtained from the primary clarifier effluents.**
(XLS)

## References

[1] WIN (2000).

[2] Pant D, Van Bogaert G, Diels L, Vanbroekhoven K (2010). A review of the substrates used in microbial fuel cells (MFCs) for sustainable energy production.. Biores Technol.

[3] Rulkens W (2008). Sewage sludge as a biomass resource for the production of energy: Overview and assessment of the various options.. Energy Fuels.

[4] Huang LP, Logan BE (2008). Electricity generation and treatment of paper recycling wastewater using a microbial fuel cell.. Appl Microbiol Biotech.

[5] Rozendal RA, Hamelers HV, Rabaey K, Keller J, Buisman CJ (2008). Towards practical implementation of bioelectrochemical wastewater treatment.. Trends Biotechnol.

[6] Logan BE, Hamelers B, Rozendal R, Schroder U, Keller J (2006). Microbial fuel cells: methodology and technology.. Environ Sci Technol.

[7] Rabaey K, Verstraete W (2005). Microbial fuel cells: novel biotechnology for energy generation.. Trends Biotechnol.

[8] Rismani-Yazdi H, Carver SM, Christy AD, Tuovinen IH (2008). Cathodic limitations in microbial fuel cells: An overview.. J Power Sources.

[9] Ishii S, Watanabe K, Yabuki S, Logan BE, Sekiguchi Y (2008). Comparison of electrode reduction activities of *Geobacter sulfurreducens* and an enriched consortium in an air-cathode microbial fuel cell.. Appl Environ Microbiol.

[10] Meyer S (2009). North City Water Reclamation Plant Annual Monitoring Report 2009.. SDRWQCB Order.

[11] Logan BE (2008). Chapter 4 Power generation. Microbial Fuel Cells.

[12] Tsujimura S, Fujita M, Tatsumi H, Kano K, Ikeda T (2001). Bioelectrocatalysis-based dihydrogen/dioxygen fuel cell operating at physiological pH.. Phys Chem Chem Phys.

[13] Di Lorenzo M, Scott K, Curtis TP, Katuri KP, Head IM (2009). Continuous feed microbial fuel cell using an air cathode and a disc anode stack for wastewater treatment.. Energy Fuels.

[14] Liu H, Ramnarayanan R, Logan BE (2004). Production of electricity during wastewater treatment using a single chamber microbial fuel cell.. Environl Sci Technol.

[15] Cheng S, Liu H, Logan BE (2006). Increased power generation in a continuous flow MFC with advective flow through the porous anode and reduced electrode spacing.. Environ Sci Technol.

[16] Ahn Y, Logan BE (2010). Effectiveness of domestic wastewater treatment using microbial fuel cells at ambient and mesophilic temperatures.. Biores Technol.

[17] Shimoyama T, Yamazawa A, Ueno Y, Watanabe K (2009). Phylogenetic analyses of bacterial communities developed in a cassette-electrode microbial fuel cell.. Microb Environ.

[18] Xing D, Cheng S, Regan JM, Logan BE (2009). Change in microbial communities in acetate- and glucose-fed microbial fuel cells in the presence of light.. Biosens Bioelectron.

[19] Liu H, Logan BE (2004). Electricity generation using an air-cathode single chamber microbial fuel cell in the presence and absence of a proton exchange membrane.. Environ Sci Technol.

[20] Cheng S, Liu H, Logan BE (2006). Increased performance of single-chamber microbial fuel cells using an improved cathode structure.. Electrochem Commun.

[21] Ishii S, Shimoyama T, Hotta Y, Watanabe K (2008). Characterization of a filamentous biofilm community established in a cellulose-fed microbial fuel cell.. BMC Microbiol.

[22] Tender LM, Reimers CE, Stecher HA, Holmes DE, Bond DR (2002). Harnessing microbially generated power on the seafloor.. Nat Biotechnol.

[23] Holmes DE, Bond DR, O'Neil RA, Reimers CE, Tender LR (2004). Microbial communities associated with electrodes harvesting electricity from a variety of aquatic sediments.. Microb Ecol.

[24] Kiely PD, Cusick R, Call DF, Selembo PA, Regan JM (2011). Anode microbial communities produced by changing from microbial fuel cell to microbial electrolysis cell operation using two different wastewaters.. Biores Technol.

[25] Chae KJ, Choi MJ, Lee JW, Kim KY, Kim IS (2009). Effect of different substrates on the performance, bacterial diversity, and bacterial viability in microbial fuel cells.. Biores Technol.

[26] Lovley DR, Holmes DE, Nevin KP (2004). Dissimilatory Fe(III) and Mn(IV) reduction.. Adv Microb Physiol.

[27] Bond DR, Holmes DE, Tender LM, Lovley DR (2002). Electrode-reducing microorganisms that harvest energy from marine sediments.. Science.

[28] Bond DR, Lovley DR (2003). Electricity production by *Geobacter sulfurreducens* attached to electrodes.. Appl Environ Microbiol.

[29] Holmes DE, Bond DR, Lovley DR (2004). Electron transfer by *Desulfobulbus propionicus* to Fe(III) and graphite electrodes.. Appl Environ Microbiol.

[30] Jung S, Regan JM (2007). Comparison of anode bacterial communities and performance in microbial fuel cells with different electron donors.. Appl Microbiol Biotechnol.

[31] Lovley DR, Giovannoni SJ, White DC, Champine JE, Phillips EJ (1993). *Geobacter metallireducens* gen. nov. sp. nov., a microorganism capable of coupling the complete oxidation of organic compounds to the reduction of iron and other metals.. Arch Microbiol.

[32] Roden EE, Lovley DR (1993). Dissimilatory Fe(III) Reduction by the marine microorganism *Desulfuromonas acetoxidans*.. Appl Environ Microbiol.

[33] Reimers CE, Girguis P, Stecher HA, Tender LM, Ryckelynck N (2006). Microbial fuel cell energy from an ocean cold seep.. Geobiology.

[34] Karlsson FH, Ussery DW, Nielsen J, Nookaew I (2011). A closer look at Bacteroides: phylogenetic relationship and genomic implications of a life in the human gut.. Microb Ecol.

[35] Gorby YA, Yanina S, McLean JS, Rosso KM, Moyles D (2006). Electrically conductive bacterial nanowires produced by *Shewanella oneidensis* strain MR-1 and other microorganisms.. PNAS.

[36] DeLong EF (1992). Archaea in coastal marine environments.. PNAS.

[37] Watanabe K, Kodama Y, Harayama S (2001). Design and evaluation of PCR primers to amplify bacterial 16 S ribosomal DNA fragments used for community fingerprinting.. J Microbiol Methods.

[38] Karlin S, Altschul SF (1990). Methods for assessing the statistical significance of molecular sequence features by using general scoring schemes.. PNAS.

[39] Colwell RK (2009). EstimateS: Statistical estimation of species richness and shared species from samples. Version 8.2. User's Guide and application.. http://purl.oclc.org/estimates.

